# Unusual occipital condyles of the skull: an osteological study with clinical implications

**DOI:** 10.1590/S1516-31802006000500009

**Published:** 2006-09-07

**Authors:** Srijit Das, Rajesh Suri, Vijay Kapur

**Keywords:** Atlanto-occipital joint, Occipital bone, Skull, Atlas, Craniofacial abnormalities, Articulação atlanto-occipital, Osso occipital, Crânio, Atlas (osso), Anormalidades craniofaciais

## Abstract

**CONTEXT:**

The occipital condyles of the skull articulate with the superior articular facets of the atlas vertebra and form an important junction between the cranium and the vertebral column. The morphological features of occipital condyles are relevant in biomechanical, anatomical and clinical studies.

**OBJECTIVE:**

To describe the anatomical profile of unusual occipital condyles detected in a bone specimen.

**CASE REPORT:**

The present osteological study provides a detailed morphological description of unusual occipital condyles showing uneven and serrated surfaces and also displaying longitudinal and transverse grooves on the left and right sides respectively. The case study also discusses the clinical importance of such anomalies. Precise anatomical knowledge of the occipital condyles is important for any craniovertebral operative procedures such as resection of the occipital condyles.

## RESUMO

**CONTEXTO:**

Os côndilos occipitais do crânio se articulam com as facetas superiores da vértebra atlas e formam uma importante junção entre o crânio e a coluna vertebral. As características morfológicas dos côndilos occipitais são relevantes em estudos biomecânicos, anatômicos e clínicos.

**OBJETIVO:**

Descrever o perfil anatômico de côndilos occipitais incomuns observados em uma amostra óssea cadavérica.

**RELATO DE CASO:**

O estudo osteológico aqui apresentado mostra a detalhada descrição morfológica de côndilos occipitais incomuns com superfícies desproporcionais e serradas, e também com sulcos longitudinais e transversais à esquerda e à direita, respectivamente. O estudo do caso também discute a importância clínica dessas anomalias. O conhecimento anatômico preciso dos côndilos occipitais é importante para qualquer procedimento cirúrgico como, por exemplo, a ressecção de côndilos occipitais.

## INTRODUCTION

The occipital condyles of the skull articulate with the superior articular facets of the atlas vertebra, thus forming the atlanto-occipital joint. Past research reports have described partition in the facets of the inferior surface of the occipital condyles.^1^ The presence of partition or double facets may interfere with the movement between the occipital condyles and the atlas vertebra.

The present case describes an interesting osteological finding on the facets of the inferior surface of the occipital condyles. An incomplete longitudinal groove and a transverse groove were observed on the facets of the occipital condyles on the left and right sides respectively.

Understanding the anatomical basis of craniovertebral anomalies is important when carrying out surgery in the region. A lateral approach during craniovertebral surgery requires resection of the occipital condyles. Hence, the morphology of the occipital condyles and their facets is important clinically.^2^

## CASE REPORT

During routine osteology teaching of the undergraduate medical students in the department of anatomy, we detected unusual occipital condyles bilaterally in an occipital bone of one cadaveric skull. The occipital condyles were carefully studied, morphometric measurements were recorded and the specimen was photographed.

### Observations

#### Left side

**Figure 1:**
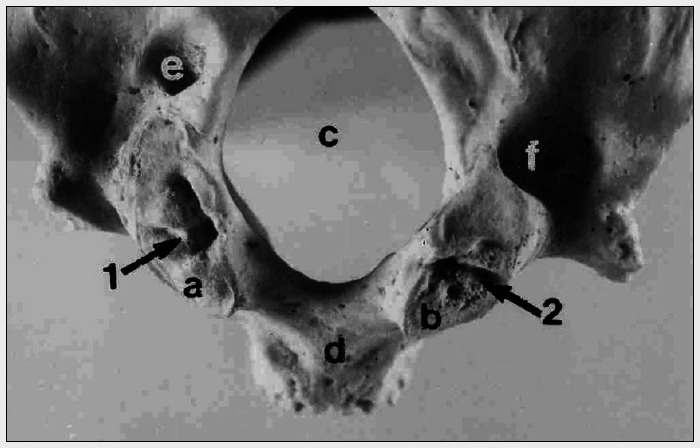
Photograph of occipital bone of a cadaveric skull (external view) showing: a. Left occipital condyle; b. Right occipital condyle; c. Foramen Magnum; d. Basiocciput; e. Left condylar canal; f. Right condylar canal; 1: Longitudinal groove on the left occipital condyle; 2: Transverse groove on the right occipital condyle.

The maximum anteroposterior and transverse dimensions of the facet measured 3 cm and 1.9 cm respectively. Multiple elevations were noted on the facet. An incomplete longitudinal groove was noticed on the medial aspect of the occipital condyle (marked as "1" in Figure 1). The surface of the facet was rough and serrated.

#### Right side

The maximum anteroposterior and transverse dimensions of the facet measured 2.5 cm and 1.7 cm respectively. Many pits and projections were noted on the facet. There were small pits on the facet and a prominent transverse groove (marked as "2" in Figure 1) traversed the facet and divided it into two parts. The surface of the facet also displayed serrations.

On both the sides, the condylar canal and the jugular foramen did not exhibit any abnormal features. No other associated anomalies were observed.

## DISCUSSION

The facets on the inferior surface of the occipital condyles of the skull are responsible for articulating with the superior articular facets of the atlas vertebra. The axes of the two occipital condyles converge anteromedially. Conventional anatomy and surgery textbooks do not describe variations in the occipital condyles. Many such anomalies of the craniovertebral region can be encountered incidentally.

Interestingly, one past study had reported constriction or division of the articular facets on the inferior surface of the occipital con- dyles.^1^ In the present case, we also observed grooves on the facets bilaterally, and the groove on the right side was placed transversely, which divided the facet into two parts. The disparity in the sizes of the left and right-sided facets and presence of bony projections and grooves provide morphological evidence of possible developmental defects. Presumably, such findings are indicative of disturbed geometrical configuration of the atlanto-occipital articulation, thereby resulting in clinical symptoms. Admittedly, the clinical history of the individual was not available to corroborate the findings.

In humans, the neural arch of the pro-atlas divides it into anterior and posterior segments, and the anterior segment forms the occipital condyles while the posterior segment forms a part of the rostral facets on the atlas vertebra.^3^ In the present case, the anomalous occipital condyles may have formed as a result of a developmental defect in the anterior segment.

The surgical treatment for any space-occupying lesion is usually performed at the level of the foramen magnum, through a ventral or dorsal approach.^2^ It has been found that the ventral approach is usually associated with more morbidity, hence the dorsal approach is usually advocated for all surgeries.^2^ Most of the surgical approaches, such as the lateral transjugular approach, transtubercular approach and transcondylar approach, require resection of the condyles.^4,5^

Understandably, surgical resection of the occipital condyles requires thorough anatomical knowledge for preoperative planning. In the present case, the articular surfaces of the facets of the occipital condyles were rough and serrated, and this may have caused disturbance to the stability and movements of the atlanto-occipital joint. The presence of bony elevations on the facet may exert pressure upon the alar ligaments, thereby altering the biomechanics of the atlanto-occipital articulation. Thorough anatomical knowledge of the anomalies of the occipital condyles may be important while performing surgery and interpreting neuroinvestigative procedures.
